# Multi-Organ Involvement in COVID-19: Beyond Pulmonary Manifestations

**DOI:** 10.3390/jcm10030446

**Published:** 2021-01-24

**Authors:** Vikram Thakur, Radha Kanta Ratho, Pradeep Kumar, Shashi Kant Bhatia, Ishani Bora, Gursimran Kaur Mohi, Shailendra K Saxena, Manju Devi, Dhananjay Yadav, Sanjeet Mehariya

**Affiliations:** 1 Department of Virology, Postgraduate Institute of Medical Education and Research (PGIMER), Chandigarh 160012, India; vik5atif@gmail.com (V.T.); ishanibora16@gmail.com (I.B.); gkmohi@gmail.com (G.K.M.); 2 Faculty of Applied Sciences and Biotechnology, Shoolini University of Biotechnology and Management Sciences, Solan 173229, India; pradeep.kumar@shooliniuniversity.com; 3 Department of Biological Engineering, College of Engineering, Konkuk University, Seoul 05029, Korea; shashikonkukuni@konkuk.ac.kr; 4 Centre for Advanced Research, Faculty of Medicine, King George’s Medical University, Lucknow 226003, India; shailen@kgmcindia.edu; 5 Department of Oral Pathology and Microbiology, RUHS College of Dental Sciences (Government Dental College), RUHS University of Rajasthan, Jaipur, Rajasthan 302016, India; bajiyamanju45@gmail.com; 6 Department of Medical Biotechnology, Yeungnam University, Gyeongsan 712-749, Korea; 7 Department of Engineering, University of Campania ‘Luigi Vanitelli’, Real Casa dell’ Annunziata, Via Roma 29, 81031 Aversa, Italy

**Keywords:** SARS-CoV-2, COVID-19, ACE-2, neurological, hepatic, dermatological, pathogenesis, therapeutics, vaccines

## Abstract

Coronavirus Disease 19 (COVID-19), due to severe acute respiratory syndrome coronavirus-2 (SARS-CoV-2) has become an on-going global health emergency affecting over 94 million cases with more than 2 million deaths globally. Primarily identified as atypical pneumonia, it has developed into severe acute respiratory distress syndrome (ARDS), a multi-organ dysfunction with associated fatality. Ever since its emergence, COVID-19 with its plethora of clinical presentations has signalled its dynamic nature and versatility of the disease process. Being a disease with droplet transmission has now assumed the proportion of a suspected airborne nature which, once proved, poses a Herculean task to control. Because of the wide distribution of the human angiotensin-converting enzyme-2 (hACE2) receptors, known for its transmission, we envisage its multiorgan spread and extensive disease distribution. Thus, an extensive review of the extrapulmonary organotropism of SARS-CoV-2 with organ-specific pathophysiology and associated manifestations like dermatological complications, myocardial dysfunction, gastrointestinal symptoms, neurologic illnesses, hepatic and renal injury is needed urgently. The plausible mechanism of site-specific viral invasion is also discussed to give a comprehensive understanding of disease complexity, to help us to focus on research priorities and therapeutic strategies to counter the disease progression. A note on the latest advancements in vaccine research will enlighten the scientific world and equip it for better preparedness.

## 1. Introduction

Coronavirus disease 2019 (COVID-19) is a novel emerging human infectious disease due to severe acute respiratory syndrome coronavirus-2 (SARS-CoV-2) first reported in Wuhan, China, in December 2019. Having been present for one year, SARS-CoV-2 had infected more than 94 million individuals with 2,031,875 deaths from 218 countries globally as of 17 January 2021 [1]. COVID-19 spread quickly across the world until a global emergency and pandemic were declared by the World Health Organization (WHO) on 30 January and 11 March 2020, respectively [2]. Untiring efforts are being invested to understand the origin, transmission, and pathogenesis of COVID-19 so that effective therapeutic agents, as well as an effective vaccine, can be developed. The R (reproductive number) for SARS-CoV-2 is estimated between 1.5–3.5 in comparison to 2.0 of SARS 2002, however, the case fatality rate (CFR) is around 2–3% in SARS-CoV-2 in comparison to 10% for SARS 2002 [3,4].

Of the seven coronaviruses (CoVs), *229E*, *NL63*, *OC43*, and *HKU1* are known for self-limiting upper respiratory tract infections [5], whereas Middle East respiratory syndrome coronavirus (MERS-CoV), SARS-CoV, and the novel SARS-CoV-2 end up with life-threatening respiratory failure and multi-organ dysfunction [6,7]. SARS-CoV-2 through spike (S) glycoproteins recognizes and binds specifically to the human angiotensin-converting enzyme 2 (hACE2) receptors expressed on type-II alveolar epithelial cells for its entry [8]. SARS-CoV-2 has a stronger binding affinity with ACE2 along with cellular transmembrane serine protease 2 (TMPRSS2) imparting virulence and aggressive properties. Following the SARS-CoV-2 binding to alveolar epithelial cells, the innate and adaptive immune system is activated leading to cytokine-release syndrome (CRS) or macrophage activation syndrome (MAS). Increased production of interleukin (IL-1, IL-6, IL-8) cytokines in plasma resulting in dyspnea, acute respiratory distress syndrome (ARDS), and death [9]. High levels of SARS-CoV-2 shedding in the upper respiratory tract, even among presymptomatic patients, is a key factor in the transmissibility of COVID-19.

## 2. Clinical Presentation and Transmission of Coronavirus Disease 2019 (COVID-19)

Because of the novel nature of the virus and lack of immunity, the presentations are dynamic and frequently changing. Being an unknown cause of atypical pneumonia, the infection gradually progressed affecting the population in Hubai province, China and thereafter, spread to different countries, the presentations varied from mild respiratory tract infection to severe pneumonia and ARDS or multi-organ dysfunction with increased mortality [10]. Other typical presentations like fever, cough, diarrhea, hemoptysis, rhinorrhea, shortness of breath, myalgia, fatigue and severe dyspnoea, lymphopenia, chest radiographic findings like ground-glass opacity are observed in COVID-19 [11]; 20% of COVID-19 patients in an older age group with pre-existing morbidities present with severe respiratory illness and ARDS whereas children and young adults have a milder illness and better prognosis [12].

The asymptomatic cases contributed to a major source of the virus and resulted in a high rate of community transmission, and thus extensive screening was required [13]. It has been estimated that up to 86% of cases with unusual presentations might have been missed in China [14]. In the beginning, COVID-19 was suspected with unusual respiratory symptoms, whereas over the progression of the pandemic involving different countries the extra-pulmonary symptoms like the neurological, cardiac, renal, gastrointestinal tract, ocular, vascular, olfactory including anosmia and ageusia were reported. The multiorgan manifestations may be correlated due to the abundancy of the ACE2 receptors in various organs [15,16,17,18] and observed with symptoms including diarrhea, poor appetite, nausea, vomiting (digestive); headache, and confusion (nervous), palmus, chest distress (cardiovascular) [19]. SARS-CoV-2 is commonly transmitted from person-to-person mainly by respiratory droplets and fomites through cough, sneeze, or by droplet inhalation and contact transmission with oral, nasal and eye, mucous membrane, including saliva.

COVID-19 diagnosis is mainly made on radiological settings like X-ray, chest computed tomography (CT) scan, and laboratory findings like lymphopenia and elevated Lactate Dehydrogenase (LDH) [12]. Nasopharyngeal and oropharyngeal swabs help in virus identification through nucleic acid detection by real-time polymerase chain reaction (RT-PCR) which is the method of choice for lab diagnosis as isolation in cell lines requires BSL-3/4 facilities [20]. Such labs are highly specialized to deal with potentially deadly infectious and exotic agents requiring the most stringent containment. The classical characteristics of BSL-4 is a full-body, air-supplied, positive pressure suit, class III biological safety cabinet, and rooms with negative pressure facility.

## 3. Cutaneous Manifestations of Severe Acute Respiratory Syndrome Coronavirus-2 (SARS-CoV-2)

Skin rashes and purpuric plaques are an interesting clinical presentation of classical coronavirus infections. The first report of the cutaneous manifestations was reported from Italy, where 20.4% (18/88) hospitalized COVID-19 patients developed an erythematous rash (14), widespread urticaria (3), and chickenpox-like vesicles (1) distributed in the trunk area [21]. In severe cases, erythematous rash, and localized or widespread urticarial rashes seem to be the most common cutaneous manifestation whereas in China only 0.2% (2/1099) confirmed COVID-19 cases had skin rashes [22].

Fernandez et al. [23] had reported a skin biopsy of a 32-year-old woman from France with COVID-19 having urticariform rash with perivascular infiltration of lymphocytes, eosinophils, and upper dermal edema on histopathology. Urticaria (1.4%) is also reported as cutaneous symptoms. A rare COVID-19 associated varicella-like papulovesicular exanthem was first observed in Italian patients by Marzano et al. [24]. Lesions were varied from scattered to diffuse with vesicular predominance in 12 (54.5%) patients with trunk and limbs involvement generally appearing 3 days after systemic symptoms. Fever, cough, headache, weakness, coryza, dyspnea, hyposmia, and hypogeusia were common systemic symptoms reported. However, SARS-CoV-2 detection in skin lesional was not performed but still represents a useful clue to suspect COVID-19 in asymptomatic patients. A dengue-like petechial rash with thrombocytopenia was reported by Joob and Wiwanitkit [25] in a COVID-19 patient from Thailand. Unusual skin manifestations like confluent erythematous-yellowish papules on both heels of a 28-year-old COVID-19 infected woman with symptoms of diarrhea, ageusia, and anosmia has been reported [26].

In a few COVID-19 patients, atopic dermatitis, and psoriasis has been aggravated as pre-existing skin disease. Joob and Wiwanitkit [27] presented an 84-year-old woman with arterial hypertension history having COVID-19 related bilateral pneumonia, later developed mild pruriginous rashes in the peri-axillary area and coalescing macules in flexural regions. Thus, various studies have reflected the possibilities of potential skin lesions of COVID-19.

## 4. Hepatic Manifestations of SARS-CoV-2

**Figure 1:**
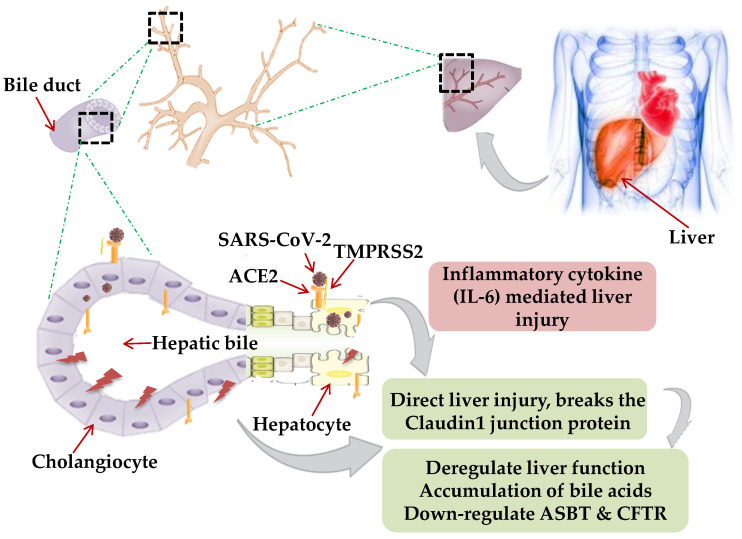
Mechanism of hepatic injury: in coronavirus disease 2019 (COVID-19) patients, hepatic injury attributed by (i) direct virus-induced cytopathic effect; (ii) virus-mediated infection-induced cytokine storm. Severe acute respiratory syndrome coronavirus-2 (SARS-CoV-2) binds to the angiotensin-converting enzyme 2 (ACE2), expressed on hepatocytes and cholangiocytes in the bile duct cells causing ablation of tight junction protein claudin 1 and down-regulation of apical sodium-dependent bile acid transporter (ASBT) and cystic fibrosis transmembrane conductance regulator (CFTR), leading to the accumulation of bile acids and contributing towards liver injury. Inflammatory cytokines (interleukin-6, IL-10, and IL-2) secretion by lymphocytes and macrophages aggravate inflammatory responses causing hepatic injury. Black dotted square frame in the figures denotes the selected area for the magnified portion.

Though primarily a respiratory pathogen, shreds of evidence indicate the liver as an extra-pulmonary site for SARS-CoV-2 infection causing liver injury ranging between 14.8% to 78% [28,29]. The possible mechanism of hepatic injury in COVID-19 could either be virus related cytopathic effect or infection-induced cytokine storm. Two independent studies on healthy cohorts by single RNA sequencing data demonstrated significant ACE2 expression (59.7%) in cholangiocytes. SARS-CoV-2 binds to the ACE2 expressed cholangiocytes and being facilitated its entry by TMPRSS2 [30]. Higher coexpression of ACE-2 and TMPRSS2 in human trophoblast cell surface antigen 2 (TROP2^high^) cholangiocyte progenitor cells of the liver has been reported [31]. The human liver ductal organoid model revealed that cholangiocyte permissiveness for SARS-CoV-2 causes direct liver injury leading to the accumulation of bile acids [32]. In 54% of COVID-19 patients, the ACE2 expression was found to be high in bile duct cells as evidenced by elevated gamma-glutamyl transferase (GGT) levels [33]. Ablation of tight junction protein claudin 1 and down-regulation of apical sodium-dependent bile acid transporter (ASBT) and cystic fibrosis transmembrane conductance regulator (CFTR) might be the contributing factors towards liver injury in COVID-19 [32] (Figure 1).

A liver biopsy of a COVID-19 positive deceased patient revealed portal inflammation with microvesicular steatosis [34]. Virus-mediated persistent activation of lymphocytes and macrophages secretes inflammatory IL-6, IL-10, IL-2, and IFN-c causing CRS and hepatic injury [35].

## 5. Cardiac Manifestations of SARS-CoV-2

Viral pathogens, especially SARS, are well-known for contributing to cardiovascular disease like acute myocarditis, acute myocardial infarction, and rapid-onset heart failure [36]. A wide range of cardiovascular events such as myocardial injury, acute coronary syndromes, cardiac arrhythmias, and heart failure are associated with COVID-19 [37,38]. In a study comprising 138 hospitalized COVID-19 patients, cardiac injury was reported in 7.2% of patients [12]. An investigation on 273 COVID-19 positive patients revealed that the higher concentration of creatine kinase isoenzyme- myocardial band (CK-MB), myohemoglobin, cardiac troponin I, and N-terminal pro-brain natriuretic peptide (NT-proBNP) in venous blood are the hallmark of heart injury [39]. A COVID-19 associated Brugada type I electrocardiographic pattern in a 61-year-old Hispanic male presented with a history of substernal chest pain, elevated CRP (150.7 mg/L) and BNP (19 pg/mL) were reported by Vidovich [40]. Sorgente et al. [41] observed an episode of supraventricular tachycardia in patients with Brugada syndrome, mostly due to plaque rupture, myocarditis, or microvascular thrombosis, resulting in virus-induced myocardial ischemia, inflammation, and ST elevation. Therefore, abnormal myocardial-associated fatalities necessitate careful monitoring of the myocardial enzyme profiles to reduce the COVID-19-associated complications in patients.

### Possible Mechanism of Cardiac Manifestations

**Figure 2:**
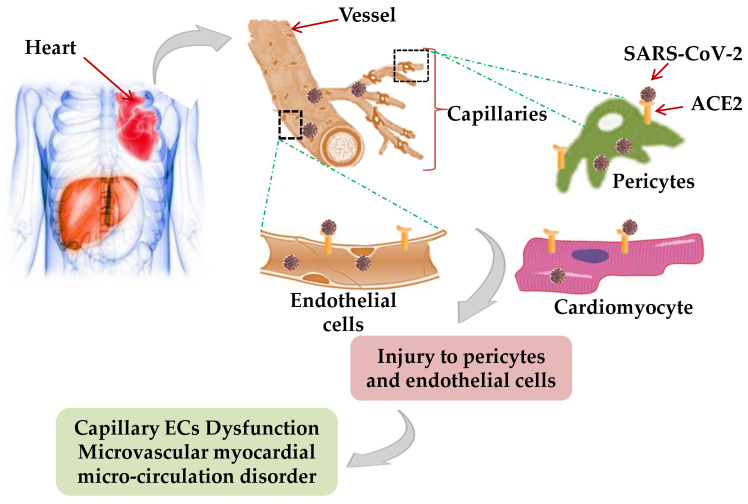
Mechanism of cardiac injury: In COVID-19 patients, SARS-CoV-2 mediated injury to cardiomyocyte, pericytes, and, capillary endothelial cells by expressing ACE2 receptor and elevating the heart susceptibility to SARS-CoV-2 infection, which induce microvascular myocardial microcirculation disorder and other cardiac abnormalities.

Single nuclear transcriptome analysis of the adult human heart identified the cardiomyocyte, endothelial cell (ECs), fibroblast, pericyte, and neuron-like cell (Neu) to be crucial for the proper functioning of the heart [42]. Moreover, the higher ACE2 expression in the cardiac pericytes illustrates the potential of SARS-CoV-2 to affect heart reflectory, indicating an intrinsic susceptibility of the heart to SARS-CoV-2 infection [43]. Therefore, during COVID-19 infection in the heart, SARS-CoV-2 mediated injury to pericytes and, capillary ECs dysfunction might induce microvascular myocardial microcirculation disorder [44] (Figure 2).

## 6. Neurological Manifestations of SARS-CoV-2

SARS-CoV and MERS-CoV have neuro-invasive properties that can assist the virus to spread from the respiratory tract to the central nervous system CNS resulting in neurological manifestations in the form of febrile seizures, convulsions, and encephalitis [45]. From the epidemiological surveys, a latency period of 7 days for SARS-CoV-2 may be enough to enter and destroy the medullary neurons. Severe destructions manifested with involuntary breathing, hyposmia, ageusia, hypoxia, symptomatic seizures, status epilepticus, nausea, and vomiting accompanied by chronic respiratory distress [17]. Patients with COVID-19 have been reported with mild (anosmia and ageusia) to severe (encephalopathy) neurological features being exacerbated by smoking, due to co-expression of hACE2 and the nicotinic acetylcholine receptor (nAChR) [46]. Recent reports of SARS-CoV-2 detection in CSF of the COVID-19 patient reasonably validate the assumption of CNS being affected by SARS-CoV-2 [47]. A COVID-19 positive patient manifested with necrotizing hemorrhagic encephalopathy evidenced through a brain CT scan [48]. Viral encephalitis, infectious toxic encephalopathy, and acute cerebrovascular disease are some of the important CNS manifestations related to COVID-19. Viral encephalitis characterized by acute onset, headache, fever, vomiting, convulsions, and consciousness disorders speculation was clinically supported by the detection of SARS-CoV-2 in the CSF of COVID-19 patients [49]. Infectious toxic encephalopathy, a reversible brain dysfunction syndrome due to systemic toxemia, and hypoxia were reported in COVID-19 patients [50]. Additionally, brain autopsies showed tissue edema and partial neuronal degeneration in deceased patients of COVID-19 infection. The SARS-CoV-2 infection has been widely reported to cause CRS, leading to acute cerebrovascular disease. Also, elevated levels of D-dimer and reduced platelet count in critically ill SARS-CoV-2 patient predispose to acute cerebrovascular events. Recent case series from China and the US describe ischaemic or hemorrhagic stroke, Guillain-Barré syndrome (GBS), or acute necrotizing encephalopathy (ANE), as neurological symptoms among COVID-19 patients [51,52] (Table 1).

### Mechanisms of Neurotropism and Neuroinvasion

**Figure 3:**
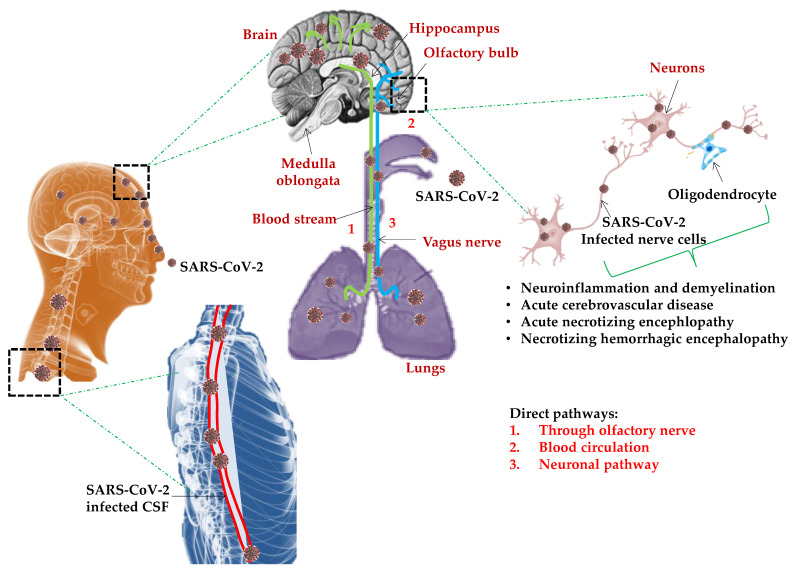
Predictable model for SARS-CoV-2 induced neurological manifestations: Numerous pathways predicted for central nervous system CNS invasion by SARS-CoV-2 to cause neuronal damage. In the olfactory neuron transport, SARS-CoV-2 can infiltrate the CNS/brain through the olfactory tract by olfactory nerves in the nasal cavity/epithelium and the olfactory bulb in the forebrain, causing inflammation and demyelinating reactions. In the hematogenous route, SARS-CoV-2 binds to the receptor ACE2 expressed in the capillary endothelium and enters the CNS by a breach in the blood–brain barrier (BBB) resulting in high blood pressure with the risk of a cerebral hemorrhage. Infected and activated macrophages, microglia, astrocytes, and glial cells in the CNS induce a pro-inflammatory state by secretion of IL-6, IL-12, and IL-15 resulting in acute necrotizing encephalopathy (ANE), hyper inflammation, and encephalopathy.

Brain infiltration through the olfactory nerves following intranasal administration could be a possibility as with *SARS-CoV* and *MERS-CoV* in the transgenic mice model. There are numerous predicted pathways for CNS invasion by SARS-CoV-2 to cause neuronal damage (Figure 3).

In the hematogenous route, SARS-CoV-2 binds to the ACE2 expressing capillary endothelium and enters the CNS by a breach in the blood–brain barrier (BBB) resulting in high blood pressure with the risk of cerebral hemorrhage. Through dynein and kinesin motor proteins, SARS-CoV-2 infects sensory or motor nerve endings by retrograde or anterograde neuronal transport as a neuronal pathway [56,57]. In the olfactory neuron transport, SARS-CoV-2 can enter the CNS/brain through the olfactory tract by olfactory nerves in the nasal cavity/epithelium and the olfactory bulb in the forebrain, causing inflammation and demyelinating reactions. Moreover, diffusion of alveolar and interstitial inflammatory exudation, results in anabolic metabolism in brain cells, leading to hypoxia and ischemic stroke. Circumventricular organs and cerebrovascular endothelial cells expressing ACE2 receptors regulate multiple neurological functions including regulation of hormone formation, the sympathoadrenal system, vascular autoregulation, and cerebral blood flow [58,59]. COVID-19 results in a large number of fatalities, mostly due to multiple organ failure induced systemic inflammatory response syndrome (SIRS). The persistence and ability of SARS-CoV-2 to infect and activate macrophages, microglia, astrocytes, and glial cells in the CNS induces a pro-inflammatory state by secretion of IL-6, IL-12, IL-15, and tumor necrosis factor-α (TNF-α) [60]. The IL-6 mediated cytokine storm, results in acute necrotizing encephalopathy (ANE) causing neuroinflammation in addition to a surge in interleukin IL-2, IL-7, interferon-γ, monocyte chemoattractant protein 1, and TNF-α leading to hyper inflammation, encephalopathy, and even stroke [61].

## 7. Renal Manifestations

Renal injury is the commonly reported COVID-19 associated renal manifestations reflecting the renal tropism of SARS-CoV-2 [62]. The burden of acute kidney injury (AKI) with COVID-19 infection was relatively low, ranging from 3–9% to as high as 15% [63,64]. Further evidence supported the renal tropism of SARS-CoV-2 by the isolation of viral RNA from urine and albuminuria and hematuria in COVID-19 infection [65,66]. Puelles et al. [67] quantified the SARS-CoV-2 viral load in autopsy tissue samples obtained from 22 COVID-19 positive deceased patients; 17 (77%) had more than two coexisting conditions, which was associated with SARS-CoV-2 tropisms for the kidneys, even in patients without a history of chronic kidney disease. Three out of six patients on autopsy had a detectable SARS-CoV-2 viral load preferentially in glomerular cells as shown in Table 2.

### The Potential Mechanisms of Renal Manifestations

In silico analysis of single-cell RNA sequencing revealed the enriched RNA expression of *ACE2*, *TMPRSS2*, and cathepsin L (*CTSL*) in podocytes and tubule epithelial cells which might facilitate the SARS-CoV-2 associated kidney injury [71,72]. ACE2 is expressed on the renal epithelial and bladder cells which counter the activation of the renin-angiotensin-aldosterone system (RAAS) [73]. SARS-CoV-2 can bind and injure the renal epithelial cells, thereby disrupting the body fluid and electrolyte homeostasis in addition to the erythropoietin and vitamin D production. Organ crosstalk (lung-kidney and heart-kidney), cytokine damage by IL-6, and systemic effects could be the underlying mechanisms for renal injury. During lung–kidney crosstalk, tubular epithelium enhances the IL-6 upregulation in serum resulting in increased alveolar-capillary permeability and pulmonary hemorrhage [74].

## 8. Gastrointestinal (GIT) Manifestations

Gastrointestinal (GIT) manifestations are revealed in 10.6% of patients with SARS and 30% of patients with MERS had diarrhea as the clinical symptoms [75]. The first case of SARS-CoV-2 infection with nausea, vomiting, and abdominal discomfort was reported from the U.S. where viral RNA was detected from the stool and respiratory specimen of a COVID-19 patient on day 7 of illness [76]. Clinically diarrhea was reported in 1–3.8% cases, diarrhea, and nausea in 10.1%, and vomiting in 3.6% [12].

Lin et al. [77] observed diarrhea and abdominal pain as evidenced in 20–50% of COVID-19 patients, which sometimes preceded respiratory symptoms. A 25-year-old female with respiratory symptoms was negative for SARS-CoV-2 in the pharyngeal aspirate but positive in the fecal sample. This might indicate feces as a source of virus transmission and the GIT region as an extrapulmonary site for virus replication [78]. Thus, early monitoring of viral RNA in the fecal specimens might benefit the disease prediction even before respiratory symptoms (Table 3).

The presence of SARS-CoV-2 in stool samples even after 11 days of viral clearance from respiratory tract samples in over half of patients indicates the alternative route of excretion of the virus [83]. Similarly, Xu et al. [84] reported 8 of the 10 infected children having persistent positive SARS-CoV-2 in rectal swabs, where nasopharyngeal swabs were negative for the virus. In a multicentric study with 1992 patients, 34% of them experienced diarrhea whereas 53% experienced one of the GIT symptoms. However 74% of the cases were mild and not associated with severe clinical course [85]. Despite the ability of SARS-CoV-2 to establish a robust infection in GIT; it might be inactivated by human colonic fluids in the intestinal lumen, hence the viral RNA transiting through GIT and shedding through the feces may not be infectious [86]. However, live SARS-CoV-2 was detected using electron microscopy in stool samples from two patients, which might focus on the potentiality of fecal transmission [87]. Even though there is evidence of GI symptoms due to SARS-CoV-*2*, its role in the disease process is yet to be demonstrated.

### Possible Mechanism for GIT Manifestations

**Figure 4:**
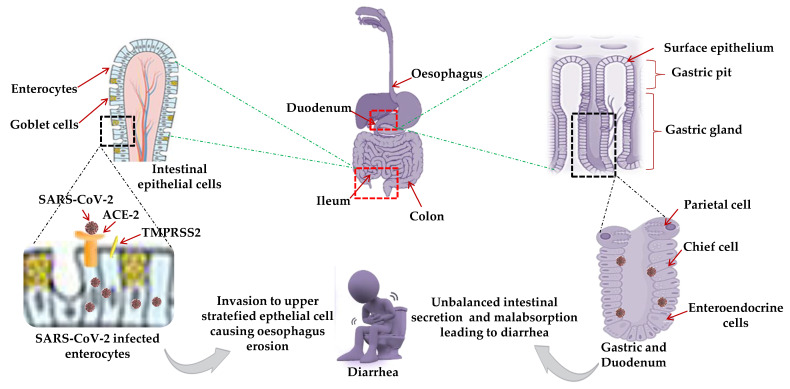
A predictive model for gastrointestinal (GIT) manifestations: High ACE2 expression and stronger binding efficiency of SARS-CoV-2 for ACE2 in absorptive intestinal epithelial cells (IECs), enterocytes in the ileum and colon, indirectly regulate intestinal inflammation and oesophageal erosion suggested the viral invasion to enterocytes of the digestive tract and stratified epithelial cells of the esophagus. High-expression of ACE2 receptors in the glandular cells of the gastric and duodenal epithelium, or proximal and distal enterocytes, leads to unbalanced intestinal secretion and mal-absorption resulting in diarrhea as a common GIT manifestation. Red dotted square frame in the figures denotes the selected area for the magnified portion.

The causative mechanism for GIT manifestation in COVID-19 positive patients is not well studied. Single-cell transcriptomic analysis of GIT (stomach, colon, ileum, and esophagus) indicated high ACE2 expression in absorptive intestinal epithelial cells (IECs) in the ileum and colon [88]. The ACE2 receptor indirectly regulates intestinal inflammation whereas TMPRSS2 is crucial for viral fusion [89]. Various hypothetical models were predicted: Higher co-expression of ACE2 and TMPRSS2 and stronger binding efficiency of SARS-CoV-2 for ACE2, located on the mature enterocytes in the ileum and colon, triggers epithelial cell fusion by TMPRSS2 and TMPRSS4 (inducing S cleavage and enhancing the fusogenic activity of the virus) suggesting the viral invasion of enterocytes of the digestive tract and stratified epithelial cells of the esophagus, resulting in oesophageal erosion [90] (Figure 4).

High expression and possible interaction of ACE2 receptors with SARS-CoV-2 in the glandular cells of gastric and duodenal epithelial cells, or in proximal and distal enterocytes, leads to unbalanced intestinal secretion and malabsorption resulting in diarrhea [91] The abundant and ubiquitous presence of ACE2 in human epithelial (small intestine) and endothelial cells might provide possible routes of transmission accounting for high transmission capacity.

## 9. Hematological Manifestations of SARS-CoV-2

Hematological parameters like alterations in immune cell population and coagulation factors in COVID-19 patients could suggest disease progression [92]. Lymphocytopenia seems to be the most common characteristic in adult patients with severe COVID-19 infection possibly due to the destruction of lymphoid organs [93]. Platelets are important in modulating inflammatory responses. A meta-analytical study showed the association of reduced platelets with COVID-19 severity, resulting in thrombocytopenia due to increased destruction or decreased platelet production [94,95]. Elevation in the levels of D-dimer is commonly observed in severe patients. Such patients are at high risk of developing thrombosis, endothelium damage, and hemorrhagic complications [96]. SARS-CoV-2 mediated endothelium injury, initiates the protective clotting cascade to minimize the internal injury, which subsequently forms undesirable internal blood clots resulting in thrombosis [97]. Angiogenesis associated ARDS was reported in the lung autopsies of 7 COVID-19 patients. It was marked by diffuse alveolar damage with perivascular T-cell infiltration and microthrombosis. Also, the severe endothelial injury was associated with intussuscepted vascular angiogenesis in the lungs [98]; 5% of large-vessel stroke incidences were reported among hospitalized patients in a single-centric study from China where coagulopathy and vascular endothelial dysfunction was the crucial presentation [52].

## 10. Unusual Manifestations of SARS-CoV-2

Subacute thyroiditis, oral lesions, large vessel stroke, rheumatologic skin disease, immune thrombocytopenia, endothelitis, pulmonary thromboembolism, pedo, and angiogenesis associated ARDS were also reported with SARS-CoV-2 as depicted in Table 4. Irregular oral lesions have been described as an early COVID-19 symptom, which needs to be proven with more pieces of evidence. Self-limited subacute thyroiditis (SAT) is characterized by neck pain, and thyroid dysfunction is usually preceded by an upper respiratory tract infection [99,100].

Previous studies showed cross-reaction of SARS-CoV-2 antigen and antibodies in patients with rheumatoid arthritis, systemic sclerosis, and systemic lupus erythematosus (SLE) [108] so there is the possibility of viral arthritis and musculoskeletal pain in COVID-19 patients, possibly due to posttranslational modification of peptides, or molecular mimicry activating T cells or epitope spreading due to T-cell associated damage by the virus leading to autoreactive T cells. SARS-CoV-2 through the ACE2 receptor directly infects the endothelial cell and facilitates the induction of endothelitis in several organs. Diffuse endothelial inflammation by host inflammatory response results in the recruitment of immune cells causing widespread endothelial dysfunction associated with apoptosis and pyroptosis. This results in shifting of vascular endothelium equilibrium towards vasoconstriction (microvascular dysfunction), resulting in inflammation with associated tissue edema, and a procoagulant state explaining the systemic impaired microcirculatory function in different vascular beds [109].

SARS-CoV-2 also directly attacks human epithelial cells of alveoli, large and small arteries, small intestine, and vascular endothelial cells. Counteracting the innate immune system activates and induces cytokine storms (IL-6) damaging the microvascular system. It activates the coagulation system while inhibiting fibrinolysis and anticoagulation systems that stimulate the liver to synthesize more thrombopoietin, fibrinogen leading to extensive thrombosis in microvessels [110]. Antiphospholipid antibodies result in endothelial injury, platelet activation, and thrombosis, with hypercoagulation. COVID-19 patients with high D-dimer levels and hypercoagulable state were associated with sudden onset of oxygen deterioration, respiratory distress, and reduced blood pressure resulting in pulmonary thromboembolism (PTE) [111].

## 11. COVID-19 in Immunocompromised Solid-Organ Transplant Recipients

Solid-organ transplant (SOT) recipients are high-risk individuals, usually on immunosuppressive therapy. Interestingly, in 2003 SARS-CoV in 2003 and 2012 MERS-CoV pandemic, SOT recipients did not appear to be associated with adverse outcomes. The role of different immunosuppressive agents such as calcineurin inhibitors and intravenous immunoglobulin (IVIG) in COVID-19 disease has not been established due to limited data on COVID-19 in transplant recipients. The typical presentation of COVID-19 in SOT recipients is the classic triad of fever, fatigue, and dry cough. A 75-year-old male and a 52-year-old female at 120 and 8 months post-transplant, respectively were diagnosed with stable graft function in males and AKI in females respectively. Extensive bilateral ground-glass opacities were the common lung abnormality reported in both cases [112].

A case of a 50-year-old COVID-19 positive man with 3rd kidney transplant recipient with IgA nephropathy induced end-stage renal disease manifested with the gastrointestinal viral disease (3–5%) and fever, further progressing to respiratory symptoms in 48 h [113]. A 39-year-old diabetic dual-organ (heart/kidney) transplant recipient positive for COVID-19 had a mild clinical course with minimal supportive care with no evidence of any graft rejection despite being on three immunosuppressive agents. The patient had additional risk factors of hypertension, diabetes mellitus, and morbid obesity, lymphopenia, elevated CRP, IL-6, D-dimer, and troponin I levels [114]. Li et al. [115] reported two heart transplant recipients with COVID-19 from Wuhan, were successfully treated, and survived.

## 12. Co-Infections with COVID-19: Viral, Bacterial, and Fungal

Diagnosing co-infections is complex owing to the clinical conditions of the infected patients [116]. During the 2003 SARS-CoV outbreak, invasive pulmonary aspergillosis was reported in only 4 among the 8422 probable SARS cases [117]. The risk of developing invasive pulmonary aspergillosis in COVID-19 patients is high as described in France where 9 out of 27 (33%) COVID-19 patients with invasive pulmonary aspergillosis admitted to an intensive care unit (ICU) [118] and 5 in Germany (26% of 19 admitted) proved in histopathology of autopsy [119]. Zhou et al. [120] showed that 50% of patients with COVID-19 died due to secondary bacterial infections. Patients with chronic obstructive pulmonary disease (COPD) will have underlying chronic bacterial infections before SARS-CoV-2 infection. Wang et al. [121] reported a case of a 37-year-old man, from Wuhan infected by SARS-CoV-2 and human immunodeficiency virus (HIV) simultaneously, highlighting the co-infection might damage T lymphocytes, impairing the immune system, B-cell dysfunction resulting in abnormal polyclonal activation and prolongation of the disease process (2 months). Chest CT showing multiple infiltrations in both lungs while nasopharyngeal swab positivity confirmed the SARS-CoV-2 infection, accompanied by dyspnea, chest pain, and palpitation. The significant decrease in the total number of immune cells i.e., B cells, T cells, and NK cells were also correlated with COVID-19 severity.

## 13. Advancements in Vaccine Research

As per the draft landscape of COVID-19 [122] dated 15 January 2021; 63 candidate vaccines are in line with clinical evaluations with 13 vaccines currently at phase 3 trial. Two mRNA vaccines i.e., 3LNP-mRNA by Pfizer-BioNTech and RNA LNP encapsulated mRNA vaccine jointly by Moderna and National Institute of Health (NIH) demonstrated an efficacy of 95% and were recently approved for the emergency use under the Emergency Use Authorization (EUA) by the US Food and Drug Administration (FDA). Another promising vaccine i.e., ChAdOx1 (chimpanzee adenovirus vaccine vector) with comparable efficacy has been developed by AstraZeneca with Oxford University, which is a non-replicating version of adenovirus containing the genetic sequence of surface spike protein which is produced after vaccination and prime the immune system to attack against the SARS-CoV-2 viral infection.

**Figure 5:**
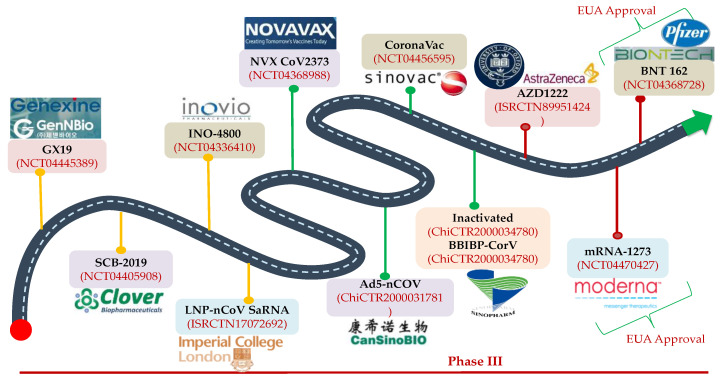
Landscape representation of COVID-19 vaccine candidates in phase III clinical trials: BNT162b2 and mRNA 1273 vaccines are in the forefront and have been granted the Emergency Use Authorization (EUA) status. Adeno based vaccine by AstraZeneca is also the promising vaccines that may be soon approved for emergency use. Other COVID-19 vaccine players are Sinovac Biotech. Ltd (Beijing, China), CasSin Biologics Inc. (Tianjin, China), Novavax (Maryland, US), Inovio (San Diego, CA, US), Clover Biopharmaceuticals (Zhejiang, China), and GenNBio Inc. (Daegu, South Korea).

The other important vaccine candidates undergoing clinical trials are the inactivated SARS-CoV-2 vaccines (Vero cells) developed by Sinopharm in collaboration with China National Biotec Group Corporation and Wuhan Institute of Biological Products, inactivated SARS-CoV-2 vaccines by Sinovac Research and Development Co. Ltd., Beijing, China and Gam-COVID-Vac Adeno-based (rAd26-S+rAdS-S) by Gamaleya Research Institute, Moscow, Russia etc. and these are depicted in Figure 5. Recently, two vaccine candidates were approved in India for clinical trials. Bharat Biotech International Limited developed an indigenous inactivated BBV152 (COVAXIN) COVID vaccine candidate with the collaborative work of the National Institute of Virology and Indian Council of Medical Research. The other one is ZyCov-D from Zydus Cadila, Ahmedabad, India which is also promising. India is also working with FluGen, Madison, USA and the University of Wisconsin-Madison, Madison, USA on an intranasal vaccine called CoroFlu with the S gene of SARS-CoV-2 insertion, built on the backbone of M2sr, a self-limiting version of influenza virus that induces immunity against COVID-19 and influenza (expressing H protein), lacking the M2 gene by restricting the replication with one cycle only. Besides, more than 173 vaccine candidates are in the pre-clinical stage. Considering the efforts, and the preliminary results from the various studies, hopefully by early 2021 we may have an approved effective vaccine available for human use to control the pandemic.

## 14. Conclusions

Since the emergence of COVID-19, the understanding of the clinical presentation of this disease is that it evolves with extrapulmonary involvement. Limited but unusual clinical cases involving the eyes, central nervous system, kidney, and liver suffice the organotropism of SARS-CoV-2. The probable explanation is the ubiquitous presence of ACE2 receptors in the various specialized cells of different organs which facilitates the binding and entry of SARS-CoV-2 inside the different cells. Unrestricted viral replication inside the cell releases the infectious virions from the cell resulting in cellular damage and eliciting the cytokine storm. The COVID-19 extra-pulmonary manifestations such as acute encephalitis in the brain, rashes on the dermis, acute renal and hepatic injury, conjunctivitis in the eyes, blood clots in the blood vessels, and loss of smell and taste are attributed by SARS-CoV-2. Understanding the different mechanisms causing organ-specific injury by SARS-CoV-2 and the route through which the virus transfers to the different locations will help the clinicians and scientists to design the treatment modality considering the critical situation of the severe COVID-19 patients. Such patients admitted to ICUs need critical monitoring of the functioning of different organs in addition to supportive oxygen therapy and antiviral administration. This will ultimately help to reduce the mortality related to COVID-19 induced acute respiratory distress syndrome and multiorgan failure until the world finds an effective and FDA-approved COVID-19 vaccine.

## Figures and Tables

**Table 1:** COVID-19 cases with neurological signs and manifestations.

Case/Study	Symptoms (Neurological)
79-year-old man (Positive-Oropharyngeal swab) [53]	Fever, acute loss of consciousness, and bilateral extensor plantar reflexes. Intracerebral hemorrhage in the right brain hemisphereIntraventricular and subarachnoid hemorrhage.
The first case of SARS-CoV-2 associated meningitis. Absence of SARS-CoV-2 RNA in nasopharyngeal swab.Positive in CSF [54]	Generalized fatigue and fever, transient generalized seizures, and neck stiffness. Convulsion accompanied by unconsciousness. Hyperintense signal in the right mesial temporal lobe in the brain along with significant paranasal sinusitis.
SARS-CoV-2 positive male with encephalitis, however, CSF is negative [55]	Fever, shortness of breath, lymphopenia, multiple subpleural ground-glass opacities in the chest, and myalgia. Progression of consciousness towards the confusion with signs of meningeal irritation.

**Table 2:** COVID-19 positive cases showing renal manifestations.

Case/Study	Symptoms (Renal)
85 COVID-19 positive patients Acute kidney injury (AKI) in 27% of patients [68]	High risk of AKI associated with age >60 years, coexisting hypertension, and coronary artery disease. Severe acute tubular injury with macrophage infiltration, and detection of viral antigen (6)
701 hospitalized patients [63]	Proteinuria (44%), Haematuria (27%), and Acute kidney injury (3.2%)
201 maintenance hemodialysis (MHD) patients. Five had COVID-19 pneumonia [69]	Diarrhea as common presenting symptoms (4/5), fever (3/5), fatigue (3/5), dyspnoea (2/5), and abdominal pain (2/5), lymphopenia (5/5), with ground-glass opacity. Fever, cough, and dyspnoea were absent.
230 haemodialysis patients; 16.1% (37) diagnosed with COVID-19 [70]	Lymphopenia, lower levels of inflammatory cytokines, and mild clinical disease

**Table 3:** Case studies showing gastrointestinal (GIT) manifestations in COVID-19 patients.

Case/Study	Symptoms (GIT/others)
26/84 COVID-19 patients with diarrhea [79]	Headache, myalgia, fatigue, cough, sputum production, nausea, vomiting
204 COVID-19 patients GIT symptoms in 99 (48.5%) Cross-sectional study, China [80]	Anorexia (83.8%) Diarrhea (29.3%)
651 COVID-19 patients 11.4% (74) patients with GI symptoms 28% lack respiratory symptoms [81]	Diarrhea, nausea, and vomiting
95 COVID-19 patients 61.1% cases with GI symptoms 52.4% (22) positive faecal samples [77]	Diarrhoea (24.2%), Nausea (17.9%), Elevated transaminases (32.6%)
1141 COVID-19 patients GIT symptoms in 16% of patients A retrospective study, China [82]	Anorexia, nausea, vomiting, diarrhea, abdominal pain

**Table 4:** Unusual manifestations of SARS-CoV-2.

Manifestation Type	Case and Studies	Presentation/Symptoms
**Oral**	45-year-old female; SARS-CoV-2 positive Nasopharyngeal swab (NPA) [101]	Irregular oral ulcer on the dorsal side of the tongue, painful inflammation of tongue papilla, macular erythematous lesion, vasculitis
**Thyroid** **(Subacute thyroiditis)**	18-year-old female positive for SARS-CoV-2; (Oropharyngeal swab) [102]	Thyroid enlargement The bilateral and diffuse hypoechoic area around the neck
**Large-vessel stroke**	5 SARS-CoV-2 positive patients (<50 years of age) [103]	New-onset symptoms of severe large-vessel ischemic stroke with the mean National Institutes of Health Stroke Scale (NIHSS) score of 17. Partial infarction of the right middle cerebral artery with a partially occlusive thrombus in the right carotid artery. Patchy ground-glass opacities in bilateral lung apices
**Endothelitis**	71-year-old male renal transplant recipient with coronary artery disease and arterial hypertension diagnosed COVID-19 positive on post mortem analysis of transplanted kidney [104]	Viral inclusion structures in endothelial cells. Inflammatory cells and apoptotic bodies accumulation in the heart and lungs
**Immune thrombocytopenia**	10-year-old SARS-CoV-2 positive patient with severe thrombocytopenia and wet purpura [105] 65-year-old SARS-CoV-2 positive woman with hypertension and autoimmune hypothyroidism [106]	Mouth: Development of purple lesions. Petechiae concentrated on lower extremities, chest and neck and ecchymoses in the popliteal regions and shins. Fatigue, fever, cough, and abdominal discomfort. Developed lower-extremity purpura, epistaxis, subarachnoid microhemorrhage, and intracranial hemorrhage
**Pedo/axillary manifestation**	16-year-old COVID-19 positive female from Ciudad Real [107] 21% (7) critically ill COVID-19 patients with acromegaly [22]	Gait alteration of red-violet lesions, itching, pain with mild swelling of both feet, nausea, dizziness, headaches, and dry eyes. Neuropathic involvement of peripheral nervous system (neuralgia) Limb ischemic symptoms: acrops, cyanosis, plantar plaques, dry gangrene, and blood blisters on the feet and hands. Significant increase in D-dimer, fibrinogen, prothrombin time, hypercoagulation, and or disseminated intravascular coagulation causing micro thrombosis
